# Evaluation of the Relationship Between Clinical Frailty Scale (CFS) and Mortality in Geriatric Patients with Pneumonia Diagnosed in Intensive Care

**DOI:** 10.3390/medicina61050781

**Published:** 2025-04-23

**Authors:** Guler Eraslan Doganay, Melek Doganci, Mustafa Ozgur Cirik, Tarkan Ozdemir, Murat Yıldız, Mehtap Tunc, Maside Arı, Fatma Ozturk Yalcin, Derya Hosgun, Banu Çakıroglu, Oral Mentes, Azra Ozabarci

**Affiliations:** 1 Department of Anesthesiology and Reanimation, Ankara Ataturk Sanatorium Training and Research Hospital, University of Health Sciences, Ankara 06290, Turkey; melekdidik@hotmail.com (M.D.); dr.ozgurr@hotmail.com (M.O.C.); mehtaptunc@hotmail.com (M.T.); ftmozt@yahoo.co.uk (F.O.Y.); deryahosgun@gmail.com (D.H.); banu.cakiroglu@yahoo.com (B.Ç.); azraarslan@yahoo.de (A.O.); 2 Department of Pulmonology, Ankara Ataturk Sanatorium Training and Research Hospital, University of Health Sciences, Ankara 06290, Turkey; tarkanozdemir78@gmail.com (T.O.); drmuratyildiz85@gmail.com (M.Y.); masidetuten@icloud.com (M.A.); omentes@live.com (O.M.)

**Keywords:** CFS, clinical frailty scale, geriatric, pneumonia, mortality, intensive care

## Abstract

*Background and Objectives*: Frailty can represent the transitional stage between successful aging and old age in need of care; it is a guide for setting goals for regaining robust old age in the individual at risk. Frailty is associated with longer intensive care unit duration, hospital stay, and higher mortality. The aim of this study was to evaluate the relationship between mortality and frailty in geriatric patients (65 years and older) admitted to the intensive care unit with a diagnosis of pneumonia. *Materials and Methods*: In total, 478 patients were included in the study. The demographic data, such as age, gender, body mass index (BMI), Charlson comorbidity index (CCI), Clinical Frailty Scale (CFS), acute physiology and chronic health evaluation (APACHE II) scores, sequential organ failure assessment score (SOFA), invasive/noninvasive mechanical ventilator days, length of stay in the hospital and intensive care unit, inotropic requirement, and 28-day mortality, were retrospectively scanned and recorded. *Results*: Advanced age, lower BMI, higher Charlson Comorbidity index (CCI), SOFA score, and CFS increased 28-day mortality. CFS was found to be associated with 28-day mortality similar to the use of inotropic agents, prolonged MV duration, and ICU length of stay (LOS). *Conclusions*: CFS is effective in predicting 28-day mortality in geriatric patients diagnosed with pneumonia in intensive care. It also provides insights into morbidity parameters such as requirement for inotropic agents, duration of mechanical ventilation (MV), and LOS ICU.

## 1. Introduction

Aging and old age are complex phenomena. Biologically, aging is associated with molecular and cellular damage that leads to a decrease in physiological capacity and an increased risk of various diseases. This decline in capacity is not linearly related to age. While some individuals over 65 lead physically and mentally active lives, others may require significant medical support to meet their basic life needs [1]. On the other hand, reduced numbers of immune cells or dysfunctional memory cells and lymphoid organ involution in older patients may explain the increased susceptibility to infectious diseases, especially those caused by Streptococcus pneumoniae and respiratory viruses.

The elderly population (aged 65 and older) worldwide is expected to increase by 25% from 2021 to 2050, effectively doubling the elderly population [2]. A ratio of the elderly population exceeding 10% of the total population is considered an indicator of population aging [3]. With this anticipated significant increase, the early identification of risk factors and the timely addressing of clinical needs have become increasingly important in health management [4]. As the number of individuals reaching advanced ages grows, the need to subdivide the aging population into specific subgroups has risen. For this purpose, the World Health Organization (WHO) categorized the population of people aged 65 and over into three subgroups: “young elderly” (65–74 years), “middle elderly” (75–84 years), and “old elderly” (85 years and older) [5].

Frailty is defined as a reduced ability of physiological systems to cope with acute stressors due to clinically recognizable aging [6]. Frailty can represent a transitional stage between successful aging and dependence on care in old age; it serves as a guide for establishing goals for achieving robust old age in individuals at risk [4]. Frail elderly individuals are less resilient to illness and are more prone to hospitalization compared to younger individuals and non-frail older adults. In this regard, frailty is associated with an increased risk of complications from medical procedures and early mortality [5].

The increase in frailty among the elderly population also leads to a worsened response to comorbidities. Consequently, rising frailty is associated with longer durations in intensive care units, longer hospital stays, and higher mortality rates [7,8]. Identifying frailty as a target for preventive interventions for age-related diseases is essential. Frailty accounts for approximately 30% of admissions to intensive care units [5].

Frailty is not solely associated with aging but is also prevalent among patients admitted to intensive care due to critical illnesses [9]. While the severity of illness increases the risk of acute death, recent data suggest that long-term outcomes are more closely related to the patient’s prior health status [10]. Frail patients who experience critical illness face a higher risk of death [9].

The progressive age-related decline in physiological systems, combined with chronic diseases and conditions, ultimately leads to decreased functional reserve capacity. Increased vulnerability heightens the risk of adverse health events, including hospital or intensive care unit admissions and death [11,12]. The state of frailty may exhibit characteristics of reversibility, especially in its earliest stages. Lee et al. reported that frailty can vary based on negative factors (such as older age, cancer history, hospitalization events, chronic obstructive pulmonary disease, cerebrovascular disease, and osteoarthritis) and positive factors (such as higher cognitive function, absence of diabetes, higher socioeconomic status, and lack of cerebrovascular disease history) associated with the patient’s condition [13].

The aim of this study is to evaluate the relationship between frailty and mortality in geriatric patients (aged 65 and older) admitted to the intensive care unit with a diagnosis of pneumonia. Patients included in the study were classified into “young elderly” (65–74 years), “middle elderly” (75–84 years), and “old elderly” (85 years and older), and their frailty indices were categorized as non-frail (CFS 1–4), mildly/moderately frail (CFS 5–6), and severely/very severely frail (CFS 7–8) [14] (Table 1).

## 2. Material and Methods

After obtaining the approval of the Ethics Committee of Ankara Ataturk Sanatorium Training and Research Hospital dated 30 March 2023 and numbered E-53610172-799-212114999, patients who were admitted to the 3rd level anesthesiology and reanimation and chest diseases intensive care units (ICU) between 1 April 2018 and 31 December 2022 were scanned, and 500 patients aged 65 years and over with a diagnosis of pneumonia were included in the study. In total, 22 of them were excluded from the study due to missing data. A total of 478 patients were included in the study.

The demographic data such as age, gender, body mass index (BMI), Charlson comorbidity index (CCI), Clinical Frailty Scale (CFS), acute physiology and chronic health evaluation (APACHE II) scores, sequential organ failure assessment score (SOFA), invasive/noninvasive mechanical ventilator days, length of stay at the hospital and intensive care unit, inotropic requirement, and 28-day mortality were retrospectively scanned and recorded. CFS, APACHE II, and SOFA scores were assessed and recorded within the first 24 h of intensive care unit admission. Information regarding 28-day mortality was obtained from the death notification system.

Exclusion criteria:

✓ Patients hospitalized for palliative care, without a diagnosis of pneumonia,
✓ Under 65 years of age,
✓ Patients with missing data,
✓ In the event of multiple ICU admissions during a hospital stay, only the first was included in the study.

## 3. Statistical Analysis

Data analyses were performed by using SPSS for Windows, version 22.0 (SPSS Inc., Chicago, IL, USA). Whether the distribution of continuous variables was normal or not was determined by the Kolmogorov–Smirnov test. The Levene test was used for the evaluation of homogeneity of variances.

Categorical data were described as the number of cases (%). Statistical analysis differences in not-normally distributed variables between two independent groups were compared by a Mann–Whitney U test. Statistical analysis differences in not-normally distributed variables between three independent groups were compared by the Kruskal–Wallis test. Categorical variables were compared using Pearson’s chi-square test or Fisher’s exact test. Degrees of relation between variables with Spearman correlation analysis were evaluated.

Univariate and multivariate logistic regression analyses were performed to assess the association between mortality and the risk factors findings.

Univariate and multivariate linear regression analyses were performed to assess the association between NIMV days, MV days, LOS ICU and LOS H, and the risk factors findings.

First of all, one variable univariate logistic/linear regression was used with risk factors that are thought to be related with 28-day mortality. Risk factors that have *p*-value < 0.25 univariate variable logistic/linear regression were included to model on multivariable logistic/linear regression. Enter the model used in multivariable logistic/linear regression. Whether every independent variable was significant in the model was analyzed with the Wald statistic on multivariable logistic regression. Whether every independent variable was significant in the model was analyzed with the t statistic on multivariable linear regression.

A *p*-value < 0.05 was accepted as a significant level on all statistical analyses.

## 4. Results

Demographic data are shown in Table 2. The largest patient group was the young elderly group with 190 patients and 63.8% of the patients participating in the study were in the Severe Frail CFS group.

There is a negative, low statistically significant relationship between NIMV duration and CFS, CCI, APACHE II, and SOFA. There is a positive, moderately statistically significant relationship between MV Duration and CFS, APACHE II, and SOFA. There is a positive, low statistically significant relationship between MV duration and CCI (Table 3).

There is a positive, low statistically significant relationship between LOS ICU and CFS. There is a negative, low statistically significant relationship between LOS hospital and CCI. There is a negative and low statistically significant relationship between the LOS hospital and SOFA (Table 3).

Age, CFS, CCI, APACHE II, SOFA, and MV days were statistically significantly higher in those who received inotropic support compared to those who did not.

BMI, NIMV days, and LOS H were found to be statistically significantly lower in those who received inotropic support compared to those who did not. We believe that the duration of NIMV was less because the patient whose clinical condition deteriorated went to intubation. The short hospitalization period may also be related to the mortal course of this patient group (Table 4).

MV duration and LOS ICU were found to be statistically significantly higher as CFS increased. Similarly, inotropic requirement and the 28-day mortality rate were found to be significantly higher as CFS increased (Table 5).

Logistic regression analysis was applied to determine the factors that increase the risk of 28-day mortality over the patients included in the study.

Firstly, univariate logistic regression analysis was applied for the factors thought to be associated with 28-day mortality. In univariate logistic regression analysis, increase in age (odds ratio = 1.032, *p* = 0.008), decrease in BMI (odds ratio = 0.954, *p* = 0.004), increase in CCI (odds ratio = 1.612, *p* ≤ 0.001), increase in APACHE II (odds ratio = 1.167, *p* ≤ 0.001), increased SOFA (odds ratio = 2.849, *p* ≤ 0.001), and increased CFS (odds ratio = 3.520, *p* ≤ 0.001) were considered to be factors that increased the risk of 28-day mortality.

Variables with *p* < 0.25 in the univariate analysis were included in the multivariate analysis.

While 28-day mortality was included in the multivariate logistic regression analysis in order to create the model that would best explain the risk of 28-day mortality, variables with no significant relationship were excluded from the analysis. The model found in the multivariate linear regression analysis is statistically significant. According to the results of multivariate logistic regression analysis, increase in age (odds ratio = 1.021, *p* = 0.024), decrease in BMI (odds ratio = 0.947, *p* = 0.023), increase in CCI (odds ratio = 1.256, *p* = 0.013), increased SOFA (odds ratio = 2.452, *p* ≤ 0.001), and increased CFS (odds ratio = 2.533, *p* ≤ 0.001) were found to increase the risk of 28-day mortality (Table 6).

**Figure 1:**
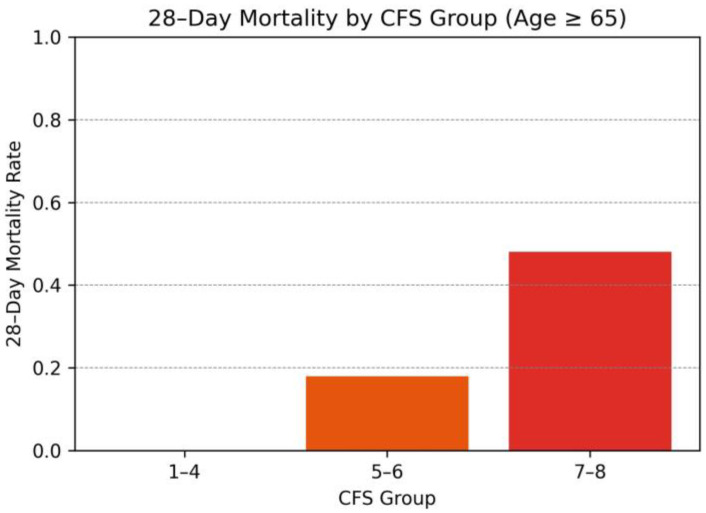
The 28-day mortality rates stratified by the Clinical Frailty Scale (CFS).

The bar graph illustrates a clear gradient in 28-day mortality across increasing frailty levels. Patients in the CFS 1–4 group exhibited the lowest mortality, while those in the CFS 7–8 group experienced the highest mortality rate. This trend highlights the prognostic significance of frailty severity, suggesting that higher CFS scores are strongly associated with poorer short-term outcomes in elderly ICU patients (Figure 1).

**Figure 2:**
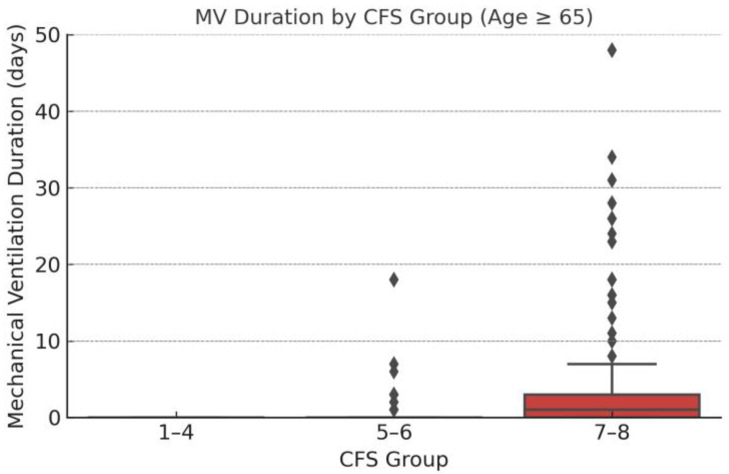
Distribution of mechanical ventilation (MV) duration across CFS groups.

The boxplot demonstrates an increase in the duration of mechanical ventilation with advancing frailty. Patients in the CFS 7–8 group required longer MV support compared to those in lower frailty categories. This observation suggests that severely frail individuals not only face higher mortality risk, but also have more prolonged intensive care needs, underscoring the clinical impact of frailty on resource utilization (Figure 2).

To assess multicollinearity among independent variables included in the multivariate logistic regression model, a variance inflation factor (VIF) analysis was performed. All VIF values were below the commonly accepted threshold of 5, indicating no critical collinearity issues among the predictors. Notably, SOFA and APACHE II scores showed slightly elevated VIFs (2.49 and 2.33, respectively), suggesting a moderate correlation that may explain why APACHE II lost statistical significance in the multivariate model despite being significant in univariate analysis (Table 7).

## 5. Discussion

This study has shown that older age, lower BMI, higher CCI, SOFA, and CFS increase 28-day mortality. CFS was found to be associated with 28-day mortality similar to (+) inotrope use, increase in MV duration, and LOS ICU. A negative correlation was found between NIMV days and CFS. We believe that the duration of NIMV was less because the patient whose clinical condition deteriorated went to intubation. The short hospitalization period may also be related to the mortal course of this patient group.

Frailty is important for understanding critical illnesses. Knowing frailty status is important in intensive care triage and management of the patient’s treatment. Assessment of frailty during ICU admission has become increasingly popular. The Clinical Frailty Scale (CFS) has proven to be a reliable tool for predicting ICU survival in intensive care patients [7,15,16].

In a study, the CFS was identified as a prognostic factor in patients aged 65 and older, demonstrating an independent association with outcomes following adjustment for relevant co-factors [17]. However, they found inconsistent associations between CFS and ICU mortality in younger patients. This difference may be sensible, because younger patients, even if they are frail, might still survive in ICU admission. They suggested that CFS scores of 5 and higher are notably effective, whereas scores of 4 and lower may influence long-term outcomes [17]. Since approximately 92% of our patients in our study had CFS 5 and above, comparisons between non-frail and frail subgroups were limited. This clustering may have constrained the statistical power to detect differences between mild and moderate frailty levels and should be considered a limitation in the interpretation of subgroup-specific results.

A study on COVID-19, examined the use of the Clinical Frailty Scale (CFS) for ICU triage in COVID-19 pneumonia, finding that CFS scores of 5 and above were good prognostic indicators for ICU admission. However, they noted that it did not support ICU admission for non-frail and mildly frail groups [18].

The World Health Organization defines frailty as the clinical condition in which older people’s ability to cope with both routine and acute stressors is diminished. Older age is often associated with, but not synonymous with, frailty [19]. In our study, while age was not found to be related to the criteria that we accept as the patient’s morbidity (MV duration, NIMV duration, LOS ICU, or LOS H), CFS was found to be proportionally related to MV duration and LOS ICU.

Additionally, the CFS score was found to be associated with 1-year mortality, regardless of age and disease. It was stated that even if the CFS score of the patients was low during their admission to the intensive care unit, they may be discharged with a higher CFS, and this could also affect mortality [20].

Likewise, a study reported associations between frailty and mortality, palliative care admissions, and the need for mechanical ventilation. It was stated that the presence of frailty along with a SOFA score greater than 2 increases the risk of mortality [8]. Similarly, in our study, we found an association between increasing CFS score and duration of mechanical ventilation (MV), requirement for inotropes, LOS ICU, and 28-day mortality. Additionally, CFS affects morbidity parameters (such as the need for inotropic support and MV duration) alongside intensive care severity scores such as APACHE II and SOFA.

In the context of multivariate modeling, the potential for collinearity among predictor variables was explored through a variance inflation factor (VIF) analysis. All included variables demonstrated VIF values below the commonly accepted threshold of 5, indicating an absence of critical multicollinearity. However, both SOFA and APACHE II scores exhibited moderately elevated VIFs (2.49 and 2.33, respectively), suggesting some degree of shared variance. This finding may account for the lack of statistical significance of APACHE II in the final multivariate logistic regression model, despite its significant association with mortality in the univariate analysis. These results underscore the importance of assessing collinearity when interpreting complex models in critically ill patient populations.

Furthermore, one study emphasized that CFS should be used as a screening tool for frailty in critically ill patients of all ages, not only in geriatric patients, due to its effective predictive ability [21].

Our study has some limitations. Firstly, the study population is small. Secondly, it is single-center. Thirdly, there is a predominance of patients with high CFS scores. Fourthly, there are challenges in assessing frailty; however, it is necessary to rely on proxies (such as family members) for scoring. The fifth limitation is that approximately 92% of patients in our study were clustered at CFS 5 and above, which may have limited the statistical power to detect differences between mild and moderate frailty levels. Lastly, younger patients were not included for comparison.

## 6. Conclusions

CFS is effective in predicting 28-day mortality in geriatric patients diagnosed with pneumonia in intensive care. It also provides insights into morbidity parameters such as requirement for inotropic agents, duration of mechanical ventilation (MV), and LOS ICU. Due to limited resources in intensive care, frailty assessment of older patients could be included in comprehensive patient assessment.

## Figures and Tables

**Table 1:** Clinical Frailty Scale (CFS) [14].

**1. Very fit:** People who are vigorous, active, and full of energy. They often exercise regularly. They are the most vigorous among their peers.	**6. Moderately frail:** completely dependent on all outside-the-home activities and household chores.
**2. Good:** No symptoms of active disease, but less vigorous than people in category 1. They exercise frequently or are occasionally very active. For example: seasonal.	**7. Severely frail:** Completely dependent on someone else for personal care for any reason (physical or cognitive). However, these are people who appear stable and are not at high risk of death (within about 6 months).
**3. Can manage well**: these are people whose medical problems are under control and who are not active other than regular walking.	**8. Very severely frail**: People who are completely dependent and are nearing the end of their lives. People who typically cannot recover from even a mild illness.
**4. Vulnerable:** Although he is not dependent on others in his daily work, his movements are limited due to disease symptoms. Their general complaints are slowness and/or feeling tired throughout the day.	**9. Terminal disease:** People who are nearing the end of their lives. This category should be applied to those with a life expectancy of less than 6 months, even without being frail.
**5. Slightly frail:** More significant slowing down of movements, needing assistance in highly instrumental daily living activities (financial issues, transfer/travel, heavy housework, and medication use). Typically, mild frailty progressively impairs ability to shop, walk outside alone, prepare meals, and do household chores.	

**Table 2:** Demographic and other data of patients.

		*n*	*%*
Age group	Young elderly (65–74)	190	39.7%
	Middle-aged (75–84)	175	36.6%
	Elderly (85 and over)	113	23.6%
Gender	Female	205	43.0%
	Male	272	57.0%
CFS group	1–4	31	7.8%
	5–6	113	28.4%
	7–8	254	63.8%
Inotropic requirement	No	347	72.6%
	Yes	131	27.4%
28-day mortality	No	257	53.8%
	Yes	221	46.2%

CFS: Clinical Frailty Scale.

**Table 3:** Correlation between risk factors and morbidity parameters of patients.

		NIMV Days	MV Days	LOS ICU	LOS H
Age	r	−0.036	−0.032	0.008	−0.001
	*p*	0.431	0.486	0.865	0.985
BMI	r	0.053	−0.063	0.020	−0.042
	*p*	0.245	0.166	0.665	0.364
CFS	r	−0.218	0.577	0.173	−0.082
	*p*	<0.001 *	<0.001 *	<0.001 *	0.075
CCI	r	−0.138	0.128	−0.041	−0.090
	*p*	0.002 *	0.005*	0.373	0.049 *
APACHE II	r	−0.177	0.349	0.056	−0.046
	*p*	<0.001 *	<0.001 *	0.225	0.319
SOFA	r	−0.292	0.499	0.055	−0.132
	*p*	<0.001 *	<0.001 *	0.229	0.004 *

r: correlation coefficient, Spearman correlation. NIMV: non-invasive mechanic ventilation, MV: mechanic ventilation, LOS ICU: length of stay in intensive care unit, LOS H: length of stay at hospital, BMI: body mass index, CFS: Clinical Frailty Scale, CCI: Charlson comorbidity index, APACHE II: acute physiology and chronic health evaluation, and SOFA: sequential organ failure assessment score. * significant *p* values.

**Table 4:** The variables according to 28-day mortality status.

	28-Day Mortality		*p*
	NO	YES	
	Med (Q1–Q3)	Med (Q1–Q3)	
Age	77.0 (70.0–82.0)	79.0 (72.0–85.0)	0.005 *
BMI	26.0 (22.2–29.3)	24.1 (21.6–27.3)	0.002 *
CFS	7.0 (6.0–7.0)	8.0 (7.0–9.0)	<0.001 *
CCI	6.0 (5.0–7.0)	7.0 (6.0–10.0)	<0.001 *
APACHE II	20.0 (17.0–24.0)	27.0 (21.0–33.0)	<0.001 *
SOFA	5.0 (5.0–6.0)	8.0 (6.0–10.0)	<0.001 *
NIMV duration	1. (0–3.0)	0 (0–1.0)	<0.001 *
MV duration	0 (0–0)	2.0 (1.0–5.0)	<0.001 *
LOS ICU	3.0 (2.0–6.0)	4.0 (2.0–8.0)	0.111
LOS H	16.0 (10.0–24.0)	11.0 (5.0–22.0)	<0.001 *

Continuous variables are expressed as median (Q1–Q3), Mann–Whitney U test *p* = level of significance, *p* < 0.05. BMI: body mass index, CFS: Clinical Frailty Scale, CCI: Charlson comorbidity index, APACHE II: acute physiology and chronic health evaluation, SOFA: sequential organ failure assessment score, NIMV: non-invasive mechanical ventilation, MV: mechanical ventilation, LOS ICU: length of stay in intensive care unit, and LOS H: length of stay at hospital. * significant *p* values.

**Table 5:** The variables according to CFS groups.

	CFS			*p*
	1–4	5–6	7–8	
	Med (Q1–Q3)	Med (Q1–Q3)	Med (Q1–Q3)	
NIMV duration	1.3 (0–3)	1.8 (0–3)	1.8 (0–2)	0.314 *^Φ^*
MV duration	0 (0–0)	0.7 (0–0)	3.8 (0–3)	<0.001 ^Φ^*
LOS ICU	3 (2–4)	4.1 (2–5)	6.9 (2–8)	0.019 ^Φ^*
LOS H	15.3 (7–21)	17.8 (10–23)	18.2 (8–24)	0.123 ^Φ^*
Inotropic requirement. *n* (%)	-	9 (8.0%)	68 (26.8%)	<0.001 ^β^*
28-day mortality. *n* (%)	0 (0%)	20 (17.7%)	122 (48.0%)	<0.001 ^β^*

Continuous variables are expressed as median (Q1–Q3) and categorical variables are expressed as either frequency (percentage). Mann–Whitney U Test *^Φ^*, chi-square test *^β^*. *p* = level of significance, *p* < 0.05 CFS: Clinical Frailty Scale, NIMV: non-invasive mechanical ventilation, MV: mechanical ventilation, LOS ICU: length of stay in intensive care unit, and LOS H: length of stay hospital. * significant *p* value.

**Table 6:** Logistic regression analysis to determine the factors affecting 28-day mortality.

Univariate Logistic Regression						Multivariate Logistic Regression				
	Wald	*p*	OR	95% CI for OR		Wald	*p*	OR	95% C.I. for OR	
				Lower	Upper				Lower	Upper
Age	7.093	0.008 *	1.032	1.008	1.056	5.088	0.024 *	1.021	1.009	1.050
Gender	1.229	0.268	1.229	0.854	1.769					
BMI	8.167	0.004 *	0.954	0.924	0.985	5.183	0.023 *	0.947	0.904	0.993
CCI	63.302	<0.001 *	1.612	1.433	1.813	6.227	0.013 *	1.256	1.050	1.502
APACHE II	77.005	<0.001 *	1.167	1.127	1.208	2.012	0.156	0.960	0.907	1.016
SOFA	103.183	<0.001 *	2.849	2.328	3.486	47.175	<0.001 *	2.452	1.898	3.166
CFS	107.597	<0.001 *	3.520	2.775	4.464	42.971	<0.001 *	2.533	1.919	3.345

Wald: test statistics, OR: odds radio, and CI: confidence interval. Hosmer–Lemeshow: *p* > 0.05. *p* = level of significance, *p* < 0.05. * significant *p* value. BMI: body mass index, CCI: Charlson comorbidity index, APACHE II: acute physiology and chronic health evaluation, SOFA: sequential organ failure assessment score, and CFS: Clinical Frailty Scale.

**Table 7:** The variance inflation factor of multivariate logistic regression analysis.

Variable	VIF Score
Age	1.40
BMI	1.01
CCI	1.53
APACHE II	2.33
SOFA	2.49
CFS	1.72

## Data Availability

All relevant data are with in the paper.
